# Right hepatectomy with preservation of the entire caudate lobe in patients with metastatic liver tumors: a case of a new hepatectomy technique and treatment strategy for patients with marginal liver function

**DOI:** 10.1186/s12893-022-01478-2

**Published:** 2022-01-15

**Authors:** Hiroyuki Kato, Yukio Asano, Masahiro Ito, Satoshi Arakawa, Norihiko Kawabe, Masahiro Shimura, Daisuke Koike, Chihiro Hayashi, Takayuki Ochi, Kenshiro Kamio, Toki Kawai, Hironobu Yasuoka, Takahiko Higashiguchi, Akihiko Horiguchi

**Affiliations:** Department of Gastroenterological Surgery, Fujita Health University School of Medicine, Bantane Hospital, 3-6-10 Otobashi Nakagawa Ward Nagoya, Aichi, 454-8509 Japan

**Keywords:** Right hepatectomy, Preservation of caudate lobe, Liver function

## Abstract

**Background:**

Performing major hepatectomy for patients with marginal hepatic function is challenging. In some cases, the procedure is contraindicated owing to the threat of postoperative liver failure. In this case report, we present the first case of marginal liver function (indocyanine green clearance retention rate at 15 min [ICGR15]: 28%) successfully treated with right hepatectomy, resulting in total caudate lobe preservation.

**Case presentation:**

A 71-year-old man was diagnosed with sigmoid colon cancer with three liver metastases (S5, S7, and S8). All of metastatic lesions shrunk after chemotherapy, but his ICGR15 and indocyanine green clearance rate (ICGK) were 21% and 0.12, respectively. Moreover, the remnant liver volume was only 39%. Therefore, portal venous embolism (PVE) of the right portal vein was suggested. Portography showed divergence of the considerably preserved right caudate lobe branch (PV1R) from the root of the right portal vein. The liver function was reevaluated 18 days after PVE was suggested. During this time, the ICGR15 (21–28%) and ICGK rate (0.12–0.10) deteriorated. The right caudate lobe was significantly enlarged; thus, a total caudate lobe-preserving hepatectomy (TCPRx) was performed. Patients eligible for TCPRx included those with (1) hepatocellular carcinoma or metastatic liver cancer, (2) no tumor in the caudate lobe, (3) marginal liver function (ICG Krem greater than 0.05 if TCPRx was adapted; otherwise, less than 0.05) and Child–Pugh classification category A, and (4) preserved PV1R and right caudate bile duct branch. The procedure was performed through (A) precise estimation of the remnant liver volume preoperatively, (B) repeated intraoperative cholangiography to confirm the biliary branch of the right caudate lobe (B1R) conservation, and (C) stapler division of posterior and anterior Glisson’s pedicles laterally to avoid injuries to the PV1R and B1R.

**Conclusions:**

Right hepatectomy with total caudate lobe preservation, following PVE, was a safe and viable surgical technique for patients with marginal liver function.

## Background

Conducting major hepatectomy in patients with marginal hepatic function is challenging. In some cases, it has been contraindicated due to the threat of postoperative liver failure. Based on the Makuuchi criteria [1], which is the gold standard for determining the appropriate hepatectomy, only segmentectomy or partial resection are recommended for patients with an indocyanine green clearance retention rate at 15 min (ICGR15) of 20% or more. However, these types of hepatectomy were reportedly more difficult to perform than lobectomy, especially in cases, involving multiple liver tumors or tumors located in the central part of the liver parenchyma. In this study, we report a new right hepatectomy technique, which preserves the entire caudate lobe. It was safely performed in a patient who underwent portal venous embolism (PVE) for multiple liver metastases secondary to colon cancer with an ICGR15 of 28%. This was the first case report to document a complete right hepatectomy with total caudate lobe preservation following PVE.

## Case presentation

**Fig. 1:**
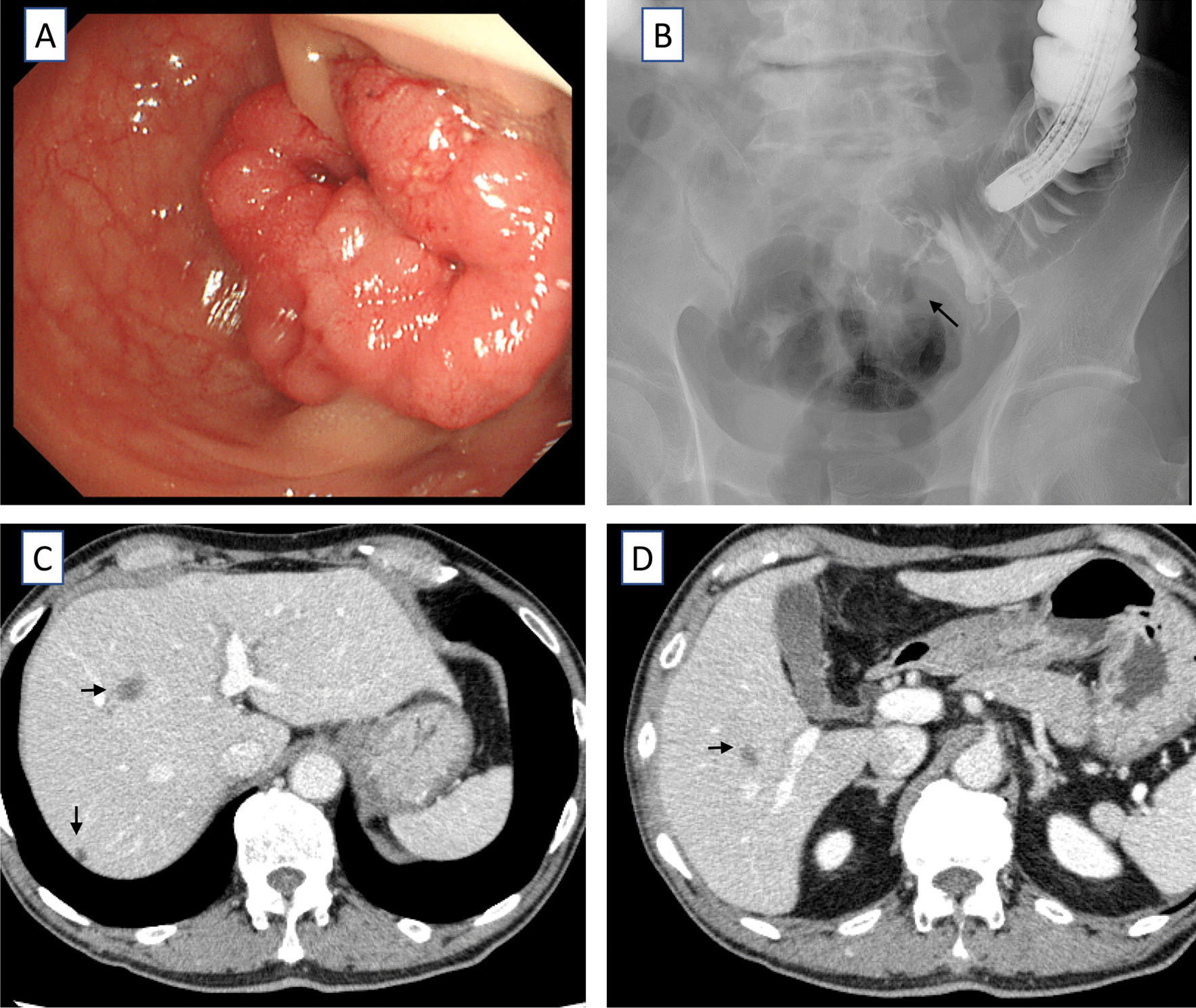
Imaging studies before portal venous embolism was suggested. **A** Colonoscopy showing the circumferential sigmoid colon cancer. **B** Findings of barium enema showing an apple core sign. **C**, **D** Enhanced computed tomography (CT) showing S8, S7, and S5 metastatic tumors

**Fig. 2:**
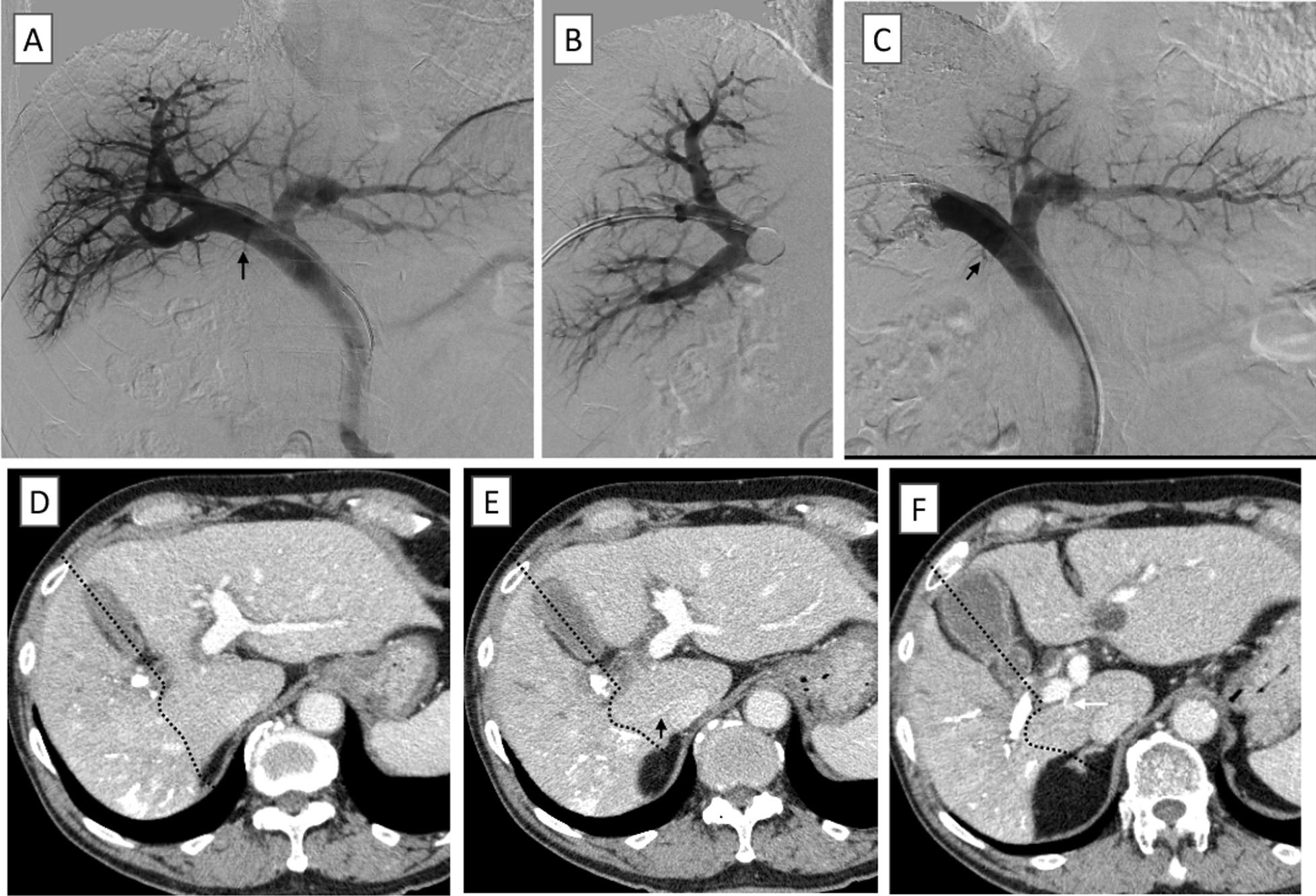
Findings of portgraphies during portal venous embolism (PVE) and enhanced CT findings 18 days after PVE suggestion. **A** Portography showing the right caudate lobe branch of the portal vein (PV1R) branching from the root of the right portal vein. **B** Imaging showing portal venous embolism (PVE). **C** Portography after PVE showing PV1R is preservable. **D**–**F** Enhanced CT on day 18 showing the enlargement of right caudate lobe (Dotted line: planned hepatic resection line, Black arrow: inferior right hepatic vein, White arrow: right caudate branch of the portal vein)

**Fig. 3:**
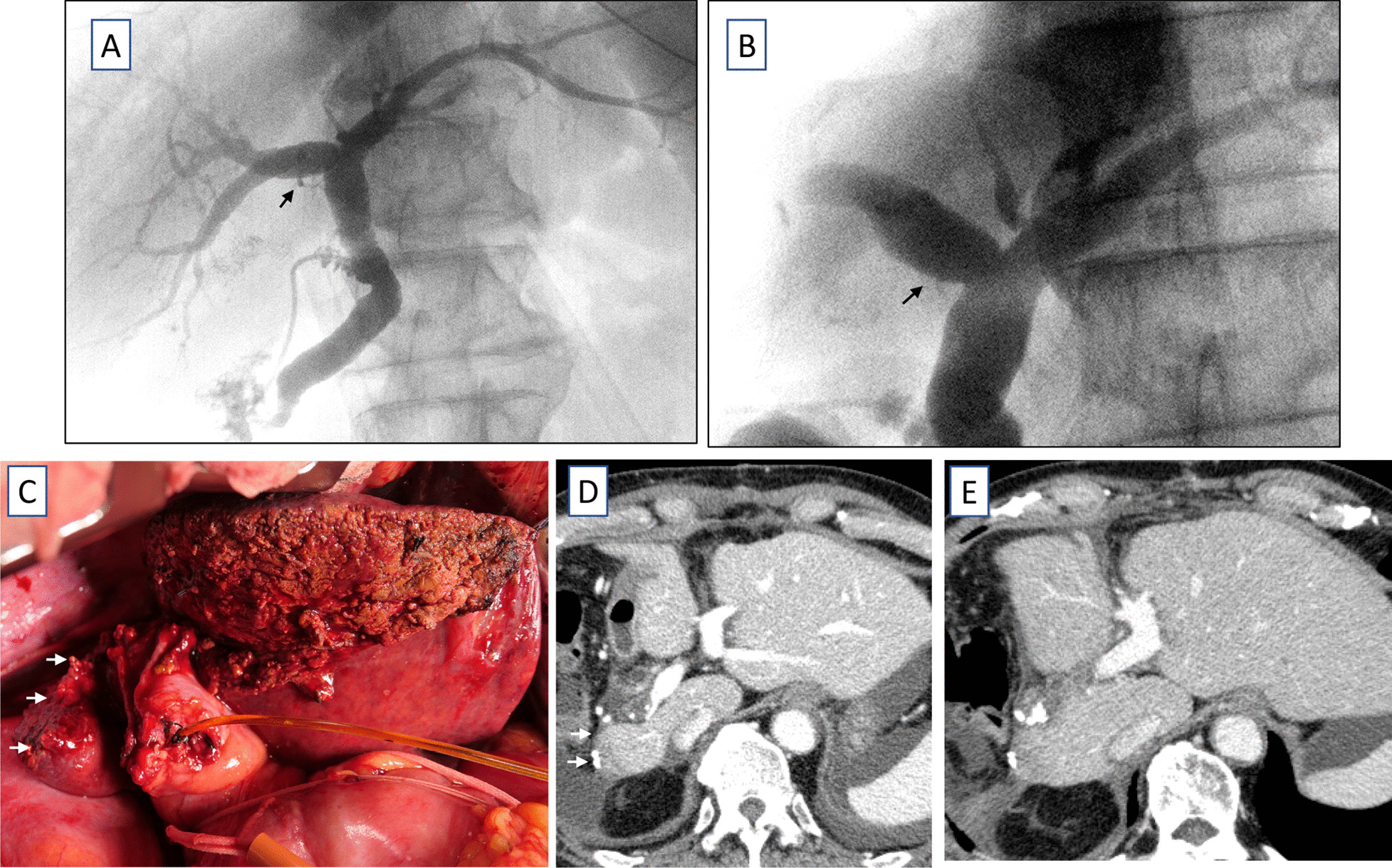
Intraoperative findings (**A**–**C**) and postoperative enhanced CT findings on postoperative day 5 and a month after surgery. **A** Intraoperative cholangiography showing the right caudate biliary branch. **B** Reevaluation of cholangiography after hepatic resection showing that the right caudate biliary branch could be preserved. **C** Intraoperative finding after hepatic resection preserved the right caudate lobe (white arrows). **D**, **E** Enhanced CT on postoperative day 7 and 1 month after surgery showing the preserved right caudate lobe (white arrows), *POD* postoperative day

A 71-year-old man was referred from the clinic due to abdominal distention. His condition was diagnosed as colon obstruction due to circumferential sigmoid colon cancer (Fig. 1A, B). He then underwent an emergent transverse colostomy, followed by sigmoid colon resection with D3 lymph node dissection. Three liver metastases were observed in S5, S7, and S8 (Fig. 1C, D). Thus, folinic acid, fluorouracil, and oxaliplatin, with bevacizumab therapy, had been given for six months before curative liver resection was performed. The chemotherapy resulted in the shrinkage of all metastatic tumors, and curative liver resection was proposed. The ICG clearance rate (ICGK) and ICGR15 were 0.12 and 21%, respectively. Meanwhile, the estimated remnant liver volume was only 39%, with a remnant ICGK value (ICGKrem) of 0.047. Therefore, a PVE of the right portal vein was suggested. Portography showed that the preserved right caudate lobe branch of the portal vein (PV1R) originated from the root of the right portal vein (Fig. 2A). Thus, PVE with PV1R preservation was performed (Fig. 2B, C). Eighteen days after PVE, liver function was reevaluated. The patient's ICGR15 (21–28%) and ICGK (0.12–0.10) were deteriorating. Moreover, computed tomography (CT) volumetry showed that the routine right hepatectomy would have resulted in a remnant liver volume (%) of 48% only (Left lobe: 493 ml/ Total liver volume: 1029 ml). Thus, it was difficult to perform a right hepatectomy due to the deteriorating liver function (ICGKrem: 0.048). The right caudate lobe was significantly enlarged (72 mL estimated by CT volumetry) because of PV1R preservation during PVE. The estimated remnant liver volume significantly increased to 55% (Left lobe with right caudate lobe: 565 ml/ total liver volume: 1029 ml) if the caudate lobe was completely preserved (Fig. 2D, E, F). Thus, a right hepatectomy with total caudate lobe preservation and concomitant colostomy closure was planned 21 days after PVE. Intraoperatively, the abdominal wall adhesions were carefully dissected. Then, the transverse colostomy was temporarily closed. The right hepatic lobe was mobilized, and the right inferior hepatic vein, which should be preserved, was exposed. Cholecystectomy and intraoperative cholangiography were performed. The right caudate branch of the bile duct diverged from the right hepatic duct (Fig. 3A). In the middle of the liver resection, the anterior and posterior Glisson’s pedicles were divided using an Endo-GIA purple cartridge. The right caudate Glisson’s pedicle was carefully preserved. The right hepatic vein was divided, and the right hepatic lobe was retrieved. After the liver resection, cholangiography was performed again to confirm the preservation of the right caudate biliary branch (Fig. 3B). The intra-abdominal findings are shown in Fig. 3C. Thereafter, the transverse colon was reconstructed using stapled functional end-to-end anastomosis. The operation time and blood loss were 424 min and 594 mL, respectively. There were no symptoms of postoperative liver failure (maximum total bilirubin level: 2.0 mg/dL on postoperative day 2) and biliary fistula. Enhanced CT scans performed on postoperative day 7 and one month after surgery revealed no findings suggestive of ischemic change, congestion, or biliary dilatation in the preserved caudate lobe, as shown in Fig. 3D and E. A surgical site infection, which was likely related to colostomy closure, occurred on postoperative day 22. However, the patient was discharged without complications one month after surgery.

## Discussion and conclusions

We report on a patient who underwent a new surgical technique, called the total caudate lobe-preserving right hepatectomy (TCPRx), despite his marginal preoperative liver function (ICGR15 of 28%). Patients eligible for TCPRx included those with (1) hepatocellular carcinoma or metastatic liver cancer, (2) no tumor in the caudate lobe, (3) marginal liver function (ICG Krem greater than 0.05 if TCPRx was adapted; otherwise, less than 0.05) and Child–Pugh classification category A, and 4) preserved PV1R and right caudate bile duct branch.

Several studies have reported quantitative measures of future remnant liver function, such as ICGR15 [2], ICG Krem [3, 4] 99mTc-galactosyl human serum albumin scintigraphy [5], and hyaluronic acid [6], to determine the indication for major hepatectomy. Moreover, biochemical parameters and clinical grading systems, such as the Child–Pugh and model for end-stage liver disease, have been categorized as passive liver function tests. These tests are not quantitative examinations. Our institution prefers using the ICG Krem value to decide the applicability of right hepatectomy. A value less than 0.05 was an indication for preoperative PVE to increase the future remnant liver volume. In this case, preoperative ICGR15 value deteriorated, likely due to the adverse effect of chemotherapy [7, 8]. The capability of PVE to maintain a tolerable remnant liver function was hypothesized. The PV1R was preserved before the PVE because there were no tumors, located in the right caudate lobe. The PV1R originated from the right portal vein, so PVE was successfully performed while preserving the PV1R. The ICGR15 and ICGK values worsened after PVE, but TCPRx was safely performed because of the favorable growth of the right caudate lobe.

As the alternative to TCPRx after PVE, anterior sectionectomy with combined partial resection of S7 was indicated in this case. However, we chose TCPRx to avoid the complexity of the latter procedure, since a prolonged operative time was expected because of the previous surgery and concomitant colon resection for stoma closure. Moreover, based on our experience and evidence from previous studies, the incidence of postoperative biliary fistula is higher with anterior sectionectomy with partial resection of S7 [9, 10]. Therefore, we selected TCPRx in this patient; however, when performing hepatic resection in patients with marginal liver function, patient safety should be the top priority and each institution should choose the method that is best suited for the patient.

TCPRx was performed through (1) precise preoperative estimation of the future remnant liver volume, (2) repeated intraoperative cholangiography to confirm the preservation of the biliary branch of the right caudate lobe (B1R), and (3) stapler division of the posterior and anterior Glisson’s pedicles laterally to avoid injuries to the PV1R and B1R.

In conclusion, right hepatectomy with total caudate lobe preservation, following PVE, was a safe and viable surgical technique for patients with multiple metastatic liver cancer and especially whose liver function is marginal. To perform this procedure, avoiding injuries to the PV1R and B1R is important especially when the posterior and anterior Glisson’s pedicles are individually divided.

## Data Availability

All the data generated or analyzed during this study are included within the article.

## Abbreviations

B1R: Biliary branch of the right caudate lobe
CT: Computed tomography
ICGR15: Indocyanine green clearance rate at 15 min
ICGK: Indocyanine green clearance rate
PV1R: Right caudate lobe branch of the portal vein
PVE: Portal venous embolism
TCPRx: Total caudate lobe-preserving right hepatectomy

## References

[1] Torzilli G, Makuuchi M, Inoue K, Takayama T, Sakamoto Y, Sugawara Y, Kubota K, Zucchi A (1999). No-mortality liver resection for hepatocellular carcinoma in cirrhotic and noncirrhotic patients: is there a way? A prospective analysis of our approach. Arch Surg.

[2] Okabe H, Beppu T, Chikamoto A, Hayashi H, Yoshida M, Masuda T, Imai K, Mima K, Nakagawa S, Kuroki H (2014). Remnant liver volume-based predictors of postoperative liver dysfunction after hepatectomy: analysis of 625 consecutive patients from a single institution. Int J Clin Oncol.

[3] Nagino M, Kamiya J, Nishio H, Ebata T, Arai T, Nimura Y (2006). Two hundred forty consecutive portal vein embolizations before extended hepatectomy for biliary cancer: surgical outcome and long-term follow-up. Ann Surg.

[4] Kobayashi Y, Kiya Y, Nishioka Y, Hashimoto M, Shindoh J (2020). Indocyanine green clearance of remnant liver (ICG-Krem) predicts postoperative subclinical hepatic insufficiency after resection of colorectal liver metastasis: theoretical validation for safe expansion of Makuuchi's criteria. HPB.

[5] Beppu T, Hayashi H, Okabe H, Masuda T, Mima K, Otao R, Chikamoto A, Doi K, Ishiko T, Takamori H (2011). Liver functional volumetry for portal vein embolization using a newly developed 99m Tc-galactosyl human serum albumin scintigraphy SPECT–computed tomography fusion system. J Gastroenterol.

[6] Nanashima A, Yamaguchi H, Shibasaki S, Sawai T, Yamaguchi E, Yasutake T, Tsuji T, Jibiki M, Nakagoe T, Ayabe H (2001). Measurement of serum hyaluronic acid level during the perioperative period of liver resection for evaluation of functional liver reserve. J Gastroenterol Hepatol.

[7] Morris-Stiff G, Tan Y-M, Vauthey JN (2008). Hepatic complications following preoperative chemotherapy with oxaliplatin or irinotecan for hepatic colorectal metastases. Eur J Surg Oncol.

[8] Fernandez FG, Ritter J, Goodwin JW, Linehan DC, Hawkins WG, Strasberg SM (2005). Effect of steatohepatitis associated with irinotecan or oxaliplatin pretreatment on resectability of hepatic colorectal metastases. J Am Coll Surg.

[9] Sakamoto K, Tamesa T, Yukio T, Tokuhisa Y, Maeda Y, Oka M (2016). Risk factors and managements of bile leakage after hepatectomy. World J Surg.

[10] Hayashi M, Hirokawa F, Miyamoto Y, Asakuma M, Shimizu T, Komeda K, Inoue Y, Arisaka Y, Masuda D, Tanigawa N (2010). Clinical risk factors for postoperative bile leakage after liver resection. Int Surg.

